# Room-temperature high-speed electrical modulation of excitonic distribution in a monolayer semiconductor

**DOI:** 10.1038/s41467-023-42568-w

**Published:** 2023-10-23

**Authors:** Guangpeng Zhu, Lan Zhang, Wenfei Li, Xiuqi Shi, Zhen Zou, Qianqian Guo, Xiang Li, Weigao Xu, Jiansheng Jie, Tao Wang, Wei Du, Qihua Xiong

**Affiliations:** 1 Jiangsu Key Laboratory for Carbon-Based Functional Materials and Devices, Institute of Functional Nano and Soft Materials (FUNSOM), Soochow University, Suzhou, 215123 PR China; 2 Key Laboratory of Mesoscopic Chemistry, School of Chemistry and Chemical Engineering, Nanjing University, Nanjing, PR China; 3 State Key Laboratory of Low-Dimensional Quantum Physics, Department of Physics, Tsinghua University, Beijing, 100084 PR China; 4 Frontier Science Center for Quantum Information, Beijing, 100084 PR China; 5 Beijing Academy of Quantum Information Sciences, Beijing, 100193 PR China; 6 Collaborative Innovation Center of Quantum Matter, Beijing, PR China

**Keywords:** Photonic devices, Optoelectronic devices and components

## Abstract

Excitons in monolayer semiconductors, benefitting from their large binding energies, hold great potential towards excitonic circuits bridging nano-electronics and photonics. However, achieving room-temperature ultrafast on-chip electrical modulation of excitonic distribution and flow in monolayer semiconductors is nontrivial. Here, utilizing lateral bias, we report high-speed electrical modulation of the excitonic distribution in a monolayer semiconductor junction at room temperature. The alternating charge trapping/detrapping at the two monolayer/electrode interfaces induces a non-uniform carrier distribution, leading to controlled in-plane spatial variations of excitonic populations, and mimicking a bias-driven excitonic flow. This modulation increases with the bias amplitude and eventually saturates, relating to the energetic distribution of trap density of states. The switching time of the modulation is down to 5 ns, enabling high-speed excitonic devices. Our findings reveal the trap-assisted exciton engineering in monolayer semiconductors and offer great opportunities for future two-dimensional excitonic devices and circuits.

## Abstract (web summary)

2D excitonic devices hold potential for on-chip optoelectronic applications. Here, the authors report high-speed in-plane electrical modulations of the excitonic distribution in monolayer semiconductor/Au electrode junctions, showing switching times as low as 5 ns at room temperature.

## Introduction

Excitons, as Coulomb-bound electron-hole pairs, are believed to be promising interconnects for bridging electrical processing and optical communication in integrated optoelectronic chips. The transport of excitons and exciton-based transistors have been well demonstrated using traditional inorganic semiconductor quantum wells^1–4^. However, the small exciton binding energy in the above system requires cryogenic temperature operation, which largely hinders their practical applications. Recently, the two-dimensional (2D) monolayer semiconductors, e.g., group VI transition metal dichalcogenides (TMDCs), with the form of MX_2_ (M = Mo, W and X = S, Se, Te etc.), have raised many research interests^5–8^. Benefitting from their atomic thickness, the quantum confinement with reduced dielectric screening leads to largely enhanced exciton binding energy (~several hundreds of meV^9–11^) in the TMDC monolayer, rendering them ideal candidates for constructing room-temperature functional excitonic devices.

To realize efficient on-chip excitonic devices, it is important to demonstrate electrical manipulation of excitons at room temperature. Usually, excitons in TMDC monolayers can be engineered by electrostatic gating with transitions between neutral excitons and charged excitons^12–15^. Correspondingly, the excitonic emission from the whole monolayer becomes brighter or darker following the changes of the doping levels. However, for excitonic circuits, it is also crucial to realize electrical manipulation of in-plane excitonic distribution or excitonic flow in the TMDC monolayer to enable optical data transfer, but with many difficulties. On one hand, the spontaneous diffusion distance of exciton is quite limited^16–20^ in TMDC monolayer mainly due to its short lifetime^21,22^. On the other hand, controlled in-plane exciton flow under lateral electric field is usually not expected considering its nature of charge neutral state. In contrast to the neutral exciton, control of charged exciton transport in TMDC monolayer with lateral electric field has been demonstrated recently, showing effective diffusion coefficient of 0.47 cm^2^/s at 6 K and a drift distance of ~100 nm under the lateral electric field of 3 × 10^4 ^V/cm^23^. However, the diffusion coefficient decreases significantly at higher temperature, as well as the drift distance, due to the enhanced trion-phonon scatterings, limiting their room-temperature applications.

Here we report electrical manipulation of excitons in an Au-WS_2_-Au junction at room temperature mediated by the bias-controlled charge trapping/detrapping at the Au/WS_2_ interface. With laterally applied DC or AC bias, the bias induced non-uniform carrier concentration in the monolayer plane further affects the spatial distribution of the excitonic compositions, i.e., populations of the neutral and charged excitons, which mimics an electrically-manipulated in-plane excitonic flow. At a local position, the excitonic emission intensity modulation increases with the increasing bias amplitude with a saturation behavior, which reflects the energetic distributions of the interface trap density of states (tDOS). The demonstrated modulation strategy is applicable to other monolayer semiconductors, such as WSe_2_ and MoS_2_, but different materials exhibit slightly different performance depending on their original doping conditions. In addition, towards high-frequency excitonic devices, we demonstrated a fast electrically-driven excitonic switch operating at frequency of 10 MHz with a high-speed switching time of 5 ns. Considering the two-terminal structure of our device with easy fabrication, room-temperature and high-speed operation, our findings may inspire new research regarding the trap-assisted opto-electronic conversion process and the development of novel 2D excitonic devices for applications in integrated optoelectronic chips.

## Results

### The Au-WS_2_-Au junction and excitonic modulation under DC bias

**Fig. 1:**
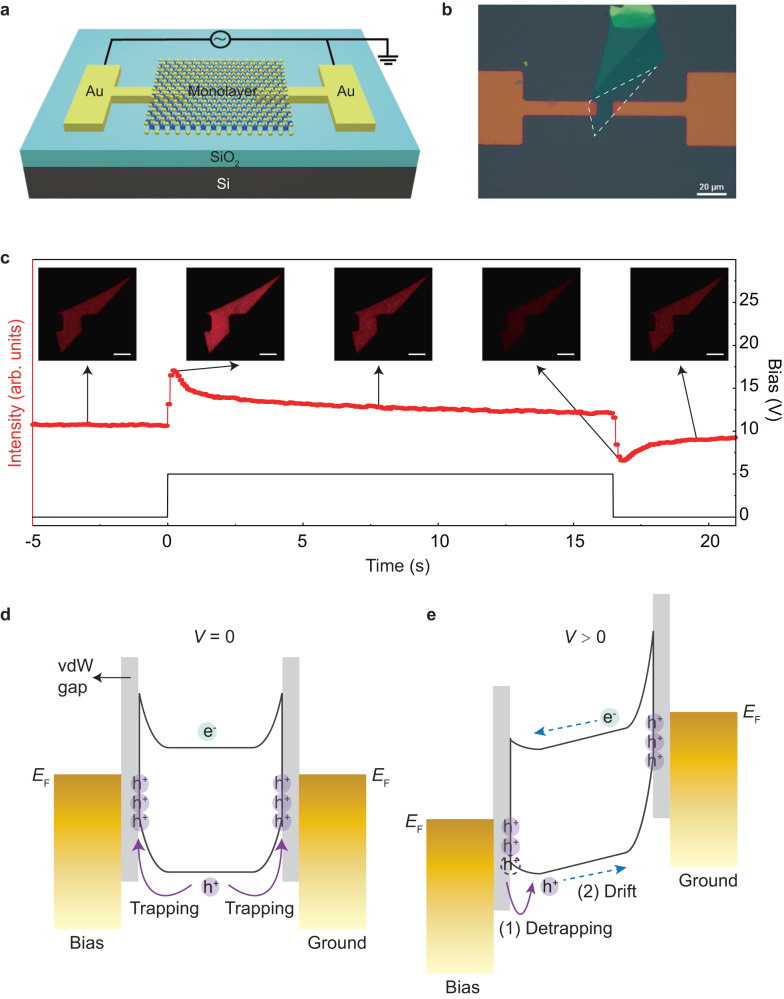
The Au-WS_2_-Au junction and modulation of excitonic emission under lateral DC bias. **a** Schematic illustration of the Au-WS_2_-Au junction. **b** Optical micrograph of the fabricated device with the monolayer boundary indicated by the white dashed line. **c** DC bias induced modulation of excitonic emission. Bias of +5 V was applied to the left electrode while the right electrode was grounded. The red line and black line indicate the excitonic emission intensity and the DC bias, respectively. The insets show the fluorescence images at specific times, where the scale bars indicate 10 μm. Energy level diagrams of the junction under zero bias (**d**) and positive bias (**e**) conditions. Bias-dependent carrier (e^-^, electrons and h^+^, holes) trapping/detrapping happens at the Au/WS_2_ interface, followed by carrier drift (blue dashed arrows) in the monolayer plane due to bias-induced band bending. *E*_F_ means the Fermi level of the Au electrode.

We prepared the Au-WS_2_-Au junction on the 285 nm SiO_2_/Si substrate using standard photolithography technique, followed by post-transfer of mechanically exfoliated monolayer semiconductors (see Methods Section). Figure 1a, b show the schematic and optical micrograph of the device, with the boundary of the WS_2_ monolayer outlined in Fig. 1b. By applying lateral DC bias (+5 V) through the two Au electrodes, we observed a clear bias modulated excitonic emission using a wide-field fluorescence microscope (see Methods Section). As shown in Fig. 1c, when bias was switched on, the excitonic emission increases abruptly and gradually decreases to a stable level afterwards. When bias was switched off, the excitonic emission decreases abruptly and recovers gradually to the original intensity level before the bias apply. Similar phenomenon was also observed under the reversed bias direction (Supplementary Movies 1, 2).

To understand our results, we referred to the energy level diagrams of the Au-WS_2_-Au junction sketched in Fig. 1d, e. The as-exfoliated WS_2_ monolayer behaves as a n-type semiconductor because of the existence of large amounts of donor-like vacancies or defects^24–26^. Correspondingly, two Schottky barriers form at the interfaces between the WS_2_ monolayer and the Au electrode. In addition to the Schottky barrier, a small van der Waals (vdW) gap^27^ may exist at the Au/WS_2_ interface, which further limits the charge transport across the interface (Supplementary Fig. 1). As a result, the photo-excited carriers, holes in this case, are trapped at the Au/WS_2_ interface at zero bias (Fig. 1d).

When bias is applied (Fig. 1e), the two Schottky barriers become asymmetric. In this case, the trapped holes at the left interface are able to tunnel into the monolayer region, and no obvious change happens at the right interface due to the finite trapping sites. As hole detrapping reduces the n-doping of the WS_2_ monolayer, more neutral excitons form, resulting in an abruptly increased excitonic emission when bias switches on. Since the hole detrapping originates from the left interface, when we just switch the bias on, the modulation is largest close to the left electrode, and we have visualized a transient state during which the monolayer shows decreasing emission intensity from the left electrode to the right electrode (Supplementary Fig. 2a, b). After that, at the constant bias window, the detrapped holes near the left interface will drift towards the right electrode according to the bias-induced band bending, leading to a constant current flow. As a result, the carrier will redistribute in the monolayer region, until reaching an equilibrium state, with uniform emission intensity in the monolayer plane close to the zero-bias condition (Supplementary Fig. 2c, d). Finally, when bias is switched off, photo-excited holes can be quickly retrapped at the left interface, resulting in increasing n-doping of the monolayer accompanied by an abrupt decreasing of excitonic emission. Meanwhile, after switching off the bias, the bias-induced band bending also disappears, thus the final relaxation is mainly caused by carrier diffusion (driven by spatially varied carrier concentration) until equilibrium is achieved. In the end, the excitonic emission recovers to the original state before bias is applied. A similar picture also applies to the negative bias with the hole trapping/detrapping occurring at the right interface (Supplementary Fig. 3).

### In-plane excitonic distribution in WS_2_ monolayer under AC bias

**Fig. 2:**
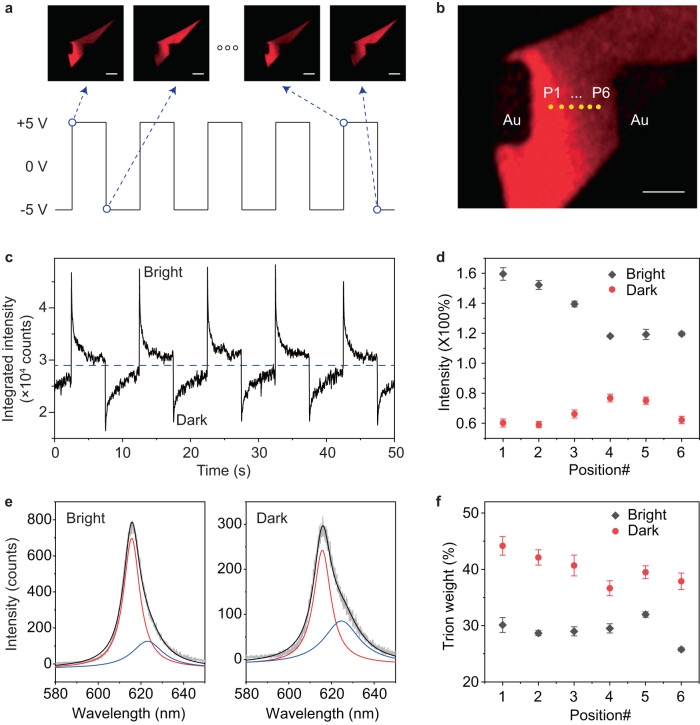
Modulation of excitonic distribution by lateral AC bias. **a** Fluorescence images of the WS_2_ monolayer under lateral AC bias, showing alternative switching between bright and dark regions at the AC frequency. The AC bias has an amplitude of ±5 V and frequency of 1 Hz. The scale bars represent 10 μm. **b** Six positions (P1 to P6) were defined in the monolayer region between the two Au electrodes. The scale bar indicates 5 μm. **c** Dynamics of excitonic emission under AC bias measured at P1 under illumination of a 532 nm CW laser. The blue dashed line indicates the intermediate emission level between the bright and dark states. **d** Position-dependent excitonic modulation by AC bias. Both the bright and dark state intensities are normalized to the intermediate emission level. **e** Emission spectra measured for the bright and dark states (at P1 position) under lateral AC bias. The two peaks (fitted with Lorentz function) correspond to the neutral exciton (red) and trion (blue) emission. **f** Position dependent trion spectral weight for the bright and dark states under lateral AC bias. For all data shown in (**c**–**f**), the AC bias has an amplitude of ±5 V and frequency of 0.1 Hz. In (**d**) and (**f**), the error bars represent for the standard deviations of measurements from 5 periods under AC bias.

Towards high-frequency electrical manipulation of excitons with non-uniform in-plane distribution, we used AC bias to simultaneously induce hole trapping/detrapping at the left and right interfaces of the Au-WS_2_-Au junction. Different from the DC case, the AC bias switches its polarity within a fast rise/fall time on the timescale of nanoseconds. Within the rise/fall time, hole trapping happens at one interface and simultaneously hole detrapping dominates at the other interface, leading to an enhanced non-uniform carrier concentration in the monolayer plane. With such effect, the wide-field fluorescence image of the WS_2_ monolayer divides into bright and dark regions which switch alternatively at the AC bias frequency (Fig. 2a) with a distinct boundary (Fig. 2b, Supplementary Movie 3).

We also investigated the excitonic modulation by AC bias via photoluminescence (PL) spectroscopy with a focused laser excitation (532 nm CW laser with the power of 50 µW, detailed in Methods Section). When exciting at a local position (P1 in Fig. 2b), the excitonic emission also switches between bright and dark states (Fig. 2c) with similar dynamics (abrupt intensity change during bias switching together with a gradual recovery during the constant bias window) as the DC bias case (Fig. 1c). The modulation depth, defined by normalizing the bright and dark state intensity to the intermediate emission intensity, is about 160% (bright) and 60% (dark) at P1. This modulation depth is found to be sensitive to the distance towards the interface as shown in Fig. 2d (Supplementary Fig. 4). Close to the interface, the modulation depth is higher (e.g., P1 and P6) for both bright and dark states, which becomes lower when moving to the monolayer center (e.g., P4).

Figure 2e presents the PL spectra for the bright and dark states at P1 position. The spectra fit well with two peaks, corresponding to the neutral exciton (red) and trion (blue) emission, respectively. The two states show different ratios between exciton and trion emission, indicating the dynamic change of carrier concentration at a local position. Further, Fig. 2f shows the trion spectral weight for the bright and dark states at P1 to P6, exhibiting a similar trend to Fig. 2d. The good correlation between the excitonic modulation depth and the trion spectral weight change (Supplementary Fig. 5) serves as strong evidence that AC bias-controlled carrier flow induces the modulation of excitonic distribution in the monolayer plane. The populations of neutral excitons and trions vary spatially according to the local electron density *N*_e_, mimicking a bias-driven excitonic flow. To be more quantitative, we have estimated *N*_e_ at P1 to P6 positions under the AC bias of ±5 V based on the intensity ratio of neutral exciton and trion peaks, which varies in the range from 1.60 × 10^13^ to 3.65 × 10^13 ^cm^−2^ (Supplementary Fig. 6). With a similar method, we also analyzed the exitonic distribution under DC bias (+5 V), which shows monotonical increasing/decreasing of trion spectral weight for the bright/dark state from P1 to P6 (Supplementary Fig. 7), and again proves that it is the charge flow leads to the excitonic flow.

### Bias-dependent excitonic modulation

The existence of trapped states is universal for almost all kinds of optoelectronic devices and plays an important role in the device performance by affecting the charge transport and recombination. The tDOS has its own spatial and energetic distributions depending on the material systems and device structures^28–31^. We resolve the energetic distribution of the interface traps in our devices via bias-dependent excitonic modulation experiments.

**Fig. 3:**
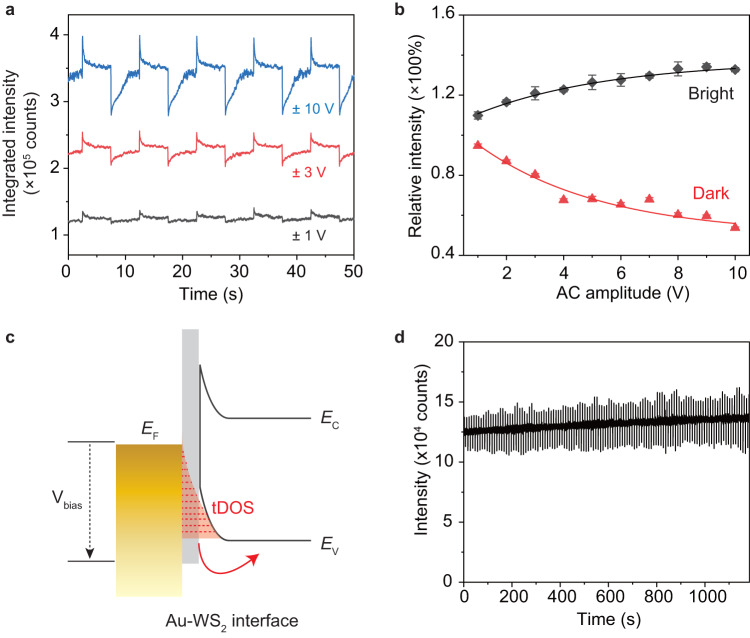
AC bias amplitude dependent excitonic modulation. **a** Dynamics of excitonic emission under AC bias of ±1 V, ±3 V, and ±10 V with frequency of 0.1 Hz. The data are shifted vertically for clarity. **b** Relative intensity of the bright and dark states as a function of the AC bias amplitude. The solid lines are fittings to the data with exponential decay functions. The error bars represent for the standard deviations of measurements from 5 periods under AC bias. **c** Sketch of the exponential-type tDOS distribution at the Au/WS_2_ interface. *E*_C_ and *E*_V_ represent the conduction band energy and valence band energy of WS_2_, respectively. *V*_bias_ is the bias applied to the Au electrode. tDOS is the abbreviation for trap density of states. **d** Stability of the excitonic modulation with a fixed AC bias over more than 1000 s.

Figure 3a shows the dynamics of excitonic emission (P1 position) measured with ±1 V, ±3 V, and ±10 V AC bias, indicating obvious bias dependency on the modulation depth. By systematically increasing the AC bias amplitude from ±1 V to ±10 V with a step of 1 V (Supplementary Fig. 8), we obtained the modulation depth as a function of the AC bias amplitude (Fig. 3b). For both bright and dark states, the modulation depth increases with the increasing bias amplitude, but with an apparent saturation at high bias. The solid lines are fittings with exponential decay function which are related to the energetic distribution of the trapped states at the Au/WS_2_ interface, featuring more shallow traps than deeper ones. The proposed energetic distribution of tDOS in our devices are sketched in Fig. 3c. At low bias, only shallow traps could be activated and contribute to the excitonic modulation. As bias increases, deeper traps start to be involved, resulting in an increased modulation depth. However, the modulation depth eventually will saturate at high bias due to the finite amounts of interface trapping sites. Further, Fig. 3d shows the dynamics of excitonic emission under a fixed AC bias amplitude. The modulation depth is stable over 1000 s, which is essential for device performance.

### Excitonic modulation based on other TMDC junctions

**Fig. 4:**
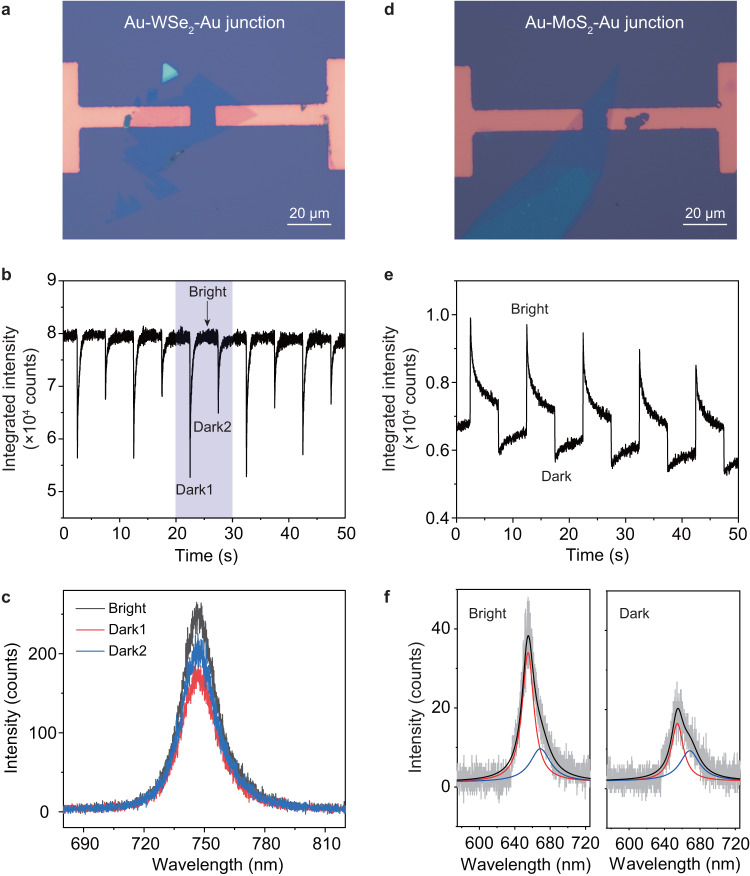
Excitonic modulation based on other TMDC junctions. **a** Optical micrography of the Au-WSe_2_-Au junction. **b** Dynamics of excitonic emission of the WSe_2_ monolayer under lateral AC bias of ±2 V and frequency of 0.1 Hz. The shaded area highlights one bright and two dark states in one period of AC bias. **c** Emission spectra of the WSe_2_ monolayer measured for the bright, dark1, and dark2 states, as indicated in (**b**). **d** Optical micrography of the Au-MoS_2_-Au junction. **e** Dynamics of excitonic emission of the MoS_2_ monolayer under lateral AC bias of ±2 V and frequency of 0.1 Hz. **f** Emission spectra of the MoS_2_ monolayer measured for the bright and dark states, as indicated in (**e**). Both spectra are fitted with two peaks, corresponding to the neutral exciton (red) and trion (blue) emission, respectively.

To demonstrate the universal applications of our strategy for excitonic modulation, we also studied junctions made with other TMDC materials such as WSe_2_ and MoS_2_. Figure 4a–c summarize the measurements from Au-WSe_2_-Au junction under AC bias of ±2 V and frequency of 0.1 Hz. The recorded excitonic emission dynamics in Fig. 4b clearly shows excitonic modulations induced by the lateral AC bias. Within each bias period, there are two dark states (labeled as dark1 and dark2) and one bright state. It means during the bias switching, no matter how the bias polarity changes, the excitonic emission intensity always decreases, which is in stark contrast to the phenomenon visualized in Au-WS_2_-Au junction, where the excitonic emission intensity increases for one bias switching direction and decreases for the opposite direction. We believe the different excitonic modulation behaviors originate from the different static doping conditions of the two monolayer semiconductors before bias is applied (Supplementary Fig. 9 and Supplementary Table 1).

WS_2_ is usually heavily n-doped, but the doping of WSe_2_ tends to be much lower^32,33^. As a result, during the AC bias switching, following the interface hole trapping/detrapping, the electron density in the WS_2_ monolayer increases or decreases accordingly, leads to suppressed or enhanced excitonic emission. However, for the WSe_2_ case, because the material is initially close to charge neutral condition, interface charge trapping and detrapping will increase the n- or p-doping of the WSe_2_ monolayer, while both will result in the suppressed excitonic emission compared to the initial charge neutral condition. Similar polarity flipping in WSe_2_ monolayer has been reported in TMDC hetero-bilayers induced by fluctuating interfacial carrier flow which happens spontaneously but randomly^34^. Here, with lateral AC bias, we achieve artificial control of the polarity flipping in WSe_2_ monolayer based on interface charge trapping/detrapping. Figure 4c shows the optical spectra for the excitonic emission at bright, dark1 and dark2 states. The single peak feature with symmetric shape implies the low charging level of WSe_2_ monolayer. Compared to the bright state, the fitted spectral linewidths for dark1 and dark2 states slightly increase from 19.6 nm to 20.5 nm and 20.3 nm. For comparison, Fig. 4d–f show the results for Au-MoS_2_-Au junction, which behaves the same way as the Au-WS_2_-Au junction owing to the *n*-doping of MoS_2_^35,36^. As shown in Fig. 4f, the optical spectra for MoS_2_ monolayer at bright and dark states (defined in Fig. 4e) again show two peaks corresponding to the neutral exciton (red) and trion (blue) emission, respectively. Compared to the bright state, the spectral weight of the trion component increases from 43.1% to 45.8% for the dark state.

### Demonstration of high-speed modulation

**Fig. 5:**
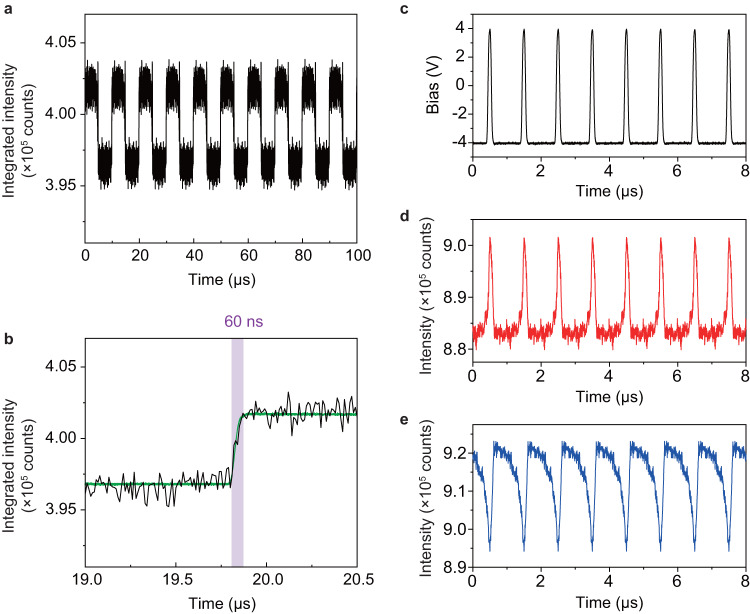
Demonstration of high-speed operation. **a** Time-resolved excitonic emission of the WS_2_ monolayer under AC bias of ±4 V and frequency of 100 KHz. **b** Zoom-in time window from panel a, showing the switching time of 60 ns (highlighted by the shaded area). The green solid line is the applied voltage profile. **c** Electrical pulses applied to the junction with the pulse width of 100 ns and the repetition frequency of 1 MHz. Time-resolved excitonic modulation induced by the applied electrical pulses measured close to the left (**d**) and right (**e**) interfaces. For all measurements, the bias was applied to the left electrode while the right electrode was grounded.

Importantly, we also demonstrated that the lateral AC bias controlled excitonic modulation features high-speed operation. As shown in Fig. 2c, although under the constant bias window, there is a slow relaxation on the order of several tens to hundreds of milliseconds (Supplementary Fig. 10), which deviates from sample to sample as it is difficult to control the monolayer/electrode and monolayer/substrate interfaces to be all the same. However, by increasing the AC bias frequency, it is possible to skip the relaxation towards the equilibrium state, while directly switching between the bright and dark states. Using the Au-WS_2_-Au junction as an example, Fig. 5a shows the time-resolved excitonic emission modulated by AC bias of ±4 V and frequency of 100 KHz. During the measurement, the CW excitation laser power was kept constant, while the time-resolved emission was collected via a time-correlated single-photon counting (TCSPC) module with synchronized photon detection and voltage apply (see Methods Section and Supplementary Fig. 11). In this case, the excitonic emission features well-separated bright and dark states with the modulation frequency controlled by the AC bias frequency. Moreover, Fig. 5b is a zoom-in of the time window from 19.0 to 20.5 μs in Fig. 5a, which clearly indicates a switching time of 60 ns.

In addition, we also verified our device could operate with the electrical pulse mode. Figure 5c shows the electrical pulses applied through the two Au electrodes with the pulse duration of 100 ns and the repetition frequency of 1 MHz. Correspondingly, Fig. 5d, e are the time-resolved excitonic modulations induced by these electrical pulses at different positions, i.e., close to the left interface (Fig. 5d) and close to the right interface (Fig. 5e), respectively. At both positions, the dynamics of excitonic emission show optical pulse features with the same repetition frequency as the electrical pulses. Moreover, the negative correlation between the two positions indicates that the bias modulated excitonic distribution in the monolayer plane is also a fast process, which responds quickly to the repeating electrical pulses.

## Discussion

To conclude, we have demonstrated the electrical modulation of excitons in monolayer semiconductors based on a unique principle. Under a lateral electric field, the bias-controlled charge trapping/detrapping at the monolayer semiconductor/metal electrode interfaces, can alter the carrier concentration and their interactions with excitons in the monolayer semiconductor, resulting in an efficient excitonic modulation. The strategy applies to different TMDC materials with the modulation profile depending on the static doping condition of the monolayer, which opens the room for further electrostatic gate control.

Because of the vdW gap in our devices, the probability for charge transport across the Au/monolayer interface is largely minimized, while the tunneling of interface charge traps into/out-of the semiconductor monolayer becomes dominant, enabling the gradient in-plane charge/exciton distribution depending on the applied bias polarity. This is in obvious contrast to previous reports^37,38^, where a lateral electric field was directly applied through the monolayer semiconductor and only induced emission quenching of the whole monolayer.

In addition to 285 nm SiO_2_/Si substrate, the lateral bias induced excitonic modulation was also obtained on the glass substrate (Supplementary Fig. 12), which further supports our trap state model, while the possible floating gate effect which might exist with the 285 nm SiO_2_/Si substrate can be completely excluded when using the glass substrate. As a result, by excluding extra capacitance from the back Si, the modulation frequency of the device on the glass substrate is further optimized to 10 MHz with a switching time down to 5 ns (Supplementary Fig. 13), which reaches the instrument response limit of our function generator. Theoretically, the upper limit of the switching speed should be determined by the RC time delay of our device, which has the room to be further optimized by shrinking the Au/WS_2_ contact area to reduce the capacitance of the junction. Meanwhile, we have also performed the excitonic modulation experiment under vacuum condition, and confirm that the modulation strategy also works well under vacuum (Supplementary Movie 4). Therefore, the heating effect accompanied with the removing or adding some residues from the atmosphere could be ruled out as the potential contribution for the excitonic modulation.

Our work shows the possibility of high-speed excitonic modulation. The experimentally measured excitonic switching time of 5 ns is orders of magnitude faster than previously reported trap-dominated (or trap-assisted) optoelectronic devices with typical temporal response on the order of millisecond to second^39–42^. This is because in our devices shallow traps can already induce a pronounced excitonic modulation. Meanwhile, under high-frequency operation, the bias polarity is quickly switched before deep traps start to be involved. Therefore, the slow relaxation process related to deep traps and band bending induced carrier drift towards equilibrium state (as shown in Figs. 2c and 3a) is skipped during high-frequency operation (Fig. 5). As a proof-of-concept, the demonstrated electrically-driven high-speed excitonic modulation at room temperature highlights the potential applications in future exciton based optoelectronic devices and interconnects.

## Methods

### Device fabrication

The Au-TMDC-Au junctions were fabricated using the following procedures. First, 20 nm thick Au electrodes (with 2 nm Cr adhesion layer) were fabricated on the 285 nm SiO_2_/Si substrate (or glass substrate) by standard UV lithography (SUSS MJB4) and electron beam evaporation (Kurt J. Lesker, PVD75). Then, mechanical exfoliation was adopted to obtain TMDC flakes from their big crystals (HQ graphene), while monolayers were identified using a fluorescence microscope. After that, the selected monolayers were aligned and transferred on top of the pre-fabricated Au electrodes using the dry transfer method based on the polydimethylsiloxane (PDMS) stamp.

### Electrical characterization

For all electrical characterizations, electrical bias was applied to the left electrode while the right electrode was grounded. I–V characterization of the junction was performed in the dark environment or under white light illumination (~80 μW/cm^2^) using a probe station (Cascade M150) equipped with a source meter (Keithley 4200) in the voltage range of −10 V to +10 V with a step of 0.05 V.

### Bias-modulated wide-field fluorescence imaging

For bias-modulated wide-field fluorescence imaging of excitonic emission (Figs. 1c, 2a, b and Supplementary Movies 1–4), the device was connected to a commercial probe station (Zhengzhou Ketan KT-Z165M4), and then the DC or AC bias was applied through a source meter (Keithley 2450) or a function generator (Tektronix AFC3011C and Rigol DG4202), respectively. A fluorescence microscope (Nikon Eclipse LV100ND) equipped with a Mercury lamp (Nikon Intensilight C-HGFI) and a charge-coupled device camera (Nikon Digital Sight DS-Fi1c) was used to record the images and movies for the excitonic emission and exciton-like flow in the WS_2_ monolayer under different bias conditions.

### Bias-modulated photoluminescence spectroscopy

For the PL time traces of excitonic emission of the Au-WS_2_-Au junctions and the corresponding PL spectra for the bright or dark states under AC bias (Figs. 2c–f, 3), a confocal PL imaging system (WITEC Alpha300 R) was used with a 532 nm CW laser (WITEC-532) as the excitation source. During the measurements, the laser was focused on the sample by an objective (Zeiss LD EC Epiplan-Neofluar Dic, 50×, NA = 0.55) and its power was kept as 50 μW. While for monolayer WSe_2_ and MoS_2_ based junctions (Fig. 4), the 633 nm CW laser (WITEC-633) was used with a power of 500 μW.

### High-speed bias-modulated excitonic emission

The high-speed modulation experiments (Fig. 5) were conducted on a home-built PL microscope system based on the Olympus microscope (BX43). During measurements, the constant 532 nm CW laser (Changchun Laser MW-ZGL-532) was focused on the WS_2_ monolayer with the objective (Olympus LMPLFLN, 50×, NA = 0.5), and the time-resolved excitonic emission under the AC bias was measured via a TCSPC module (Swabian Instruments Time Tagger Ultra) which was synchronized with the function generator (Tektronix AFC3011C and Rigol DG4202) and the single photon avalanche diode (SPAD, MPD PD-100-CTE). All experiments were conducted in ambient environment at room temperature.

### Supplementary information

Supplementary Information

Peer Review File

Description of Additional Supplementary Files

Supplementary Movie 1

Supplementary Movie 2

Supplementary Movie 3

Supplementary Movie 4

## Data Availability

The data that support the findings of this study are available from the corresponding authors upon request.
