# Supplementary material for: Room-temperature high-speed electrical modulation of excitonic distribution in a monolayer semiconductor
Source: Nat Commun. 2023 Oct 23;14:6701. doi: 10.1038/s41467-023-42568-w (PMC10593816; doi:10.1038/s41467-023-42568-w)
Supplement: Supplementary file 1 — Supplementary Information [file 41467_2023_42568_MOESM1_ESM.pdf]

Supplementary Information for

Room-temperature high-speed electrical modulation of excitonic distribution in a monolayer semiconductor

Guangpeng Zhu[1], Lan Zhang[1], Wenfei Li[1], Xiuqi Shi[1], Zhen Zou[1], Qianqian Guo[1], Xiang Li[1], Weigao Xu[2], Jiansheng Jie[1], Tao Wang[1*], Wei Du[1*], Qihua Xiong[3,4,5,6]

[1]Jiangsu Key Laboratory for Carbon-Based Functional Materials and Devices, Institute of Functional Nano and Soft Materials (FUNSOM), Soochow University, Suzhou 215123, P. R. China

[2]Key Laboratory of Mesoscopic Chemistry, School of Chemistry and Chemical Engineering, Nanjing University, Nanjing, P. R. China

[3]State Key Laboratory of Low-Dimensional Quantum Physics, Department of Physics, Tsinghua University, Beijing 100084, P.R. China

[4]Frontier Science Center for Quantum Information, Beijing 100084, P.R. China

[5]Beijing Academy of Quantum Information Sciences, Beijing 100193, P.R. China

[6]Collaborative Innovation Center of Quantum Matter, Beijing, P.R. China

*Email: duwei2021@suda.edu.cn; wangtao2019@suda.edu.cn

Supplementary Note 1

Supplementary Figure 1 shows the *I-V* characterization of the Au-WS_2-Au junction in the bias range of -10 V to +10 V. When the characterization was conducted in the dark environment, the measured current is significantly low (close to the noise level of our probe station), which shows bad charge transport across the Au-WS_2-Au junction. Such bad charge transport indicates the existence of van der Waals gaps at the Au/WS_2 interface, which has the possibility to accumulate interface traps.

When the Au-WS_2-Au junction is illuminated by white LED (~80 μW/cm^2), the measured current nearly does not change at zero bias, while, increases by almost two orders of magnitude at higher bias. This phenomenon shows the obvious photocurrent effect, and indicates the bias-induced band bending in the WS_2 monolayer which facilitates the drift of charge carriers in the monolayer plane. At the same time, the negligible photocurrent effect at zero bias also indicates that the photo-excited carriers, holes in this case, are trapped at the Au/WS_2 interface.

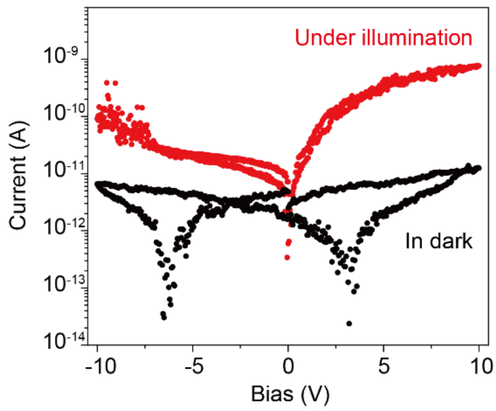

Supplementary Figure 1. *I-V* characteristics of the Au-WS_2-Au junction in the voltage range of -10 V to +10 V, measured in the dark environment (black dots) and under white light illumination (red dots).

Supplementary Note 2

Supplementary Figure 2 shows two wide-field fluorescence images recorded from the same junction under +5 V DC bias. Initially, when the bias is just switched on, the modulation is largest close to the left interface, where the hole detrapping happens, and the monolayer emission intensity decreases from left to right (Supplementary Figure 2a,b). After that, at the constant bias window, the carriers redistribute in the monolayer plane due to the band bending induced carrier drift. As a result, the modulation relaxes to an equilibrium state, which shows uniform emission in the monolayer plane (Supplementary Figure 2c,d) with intensities closer to the zero-bias condition.

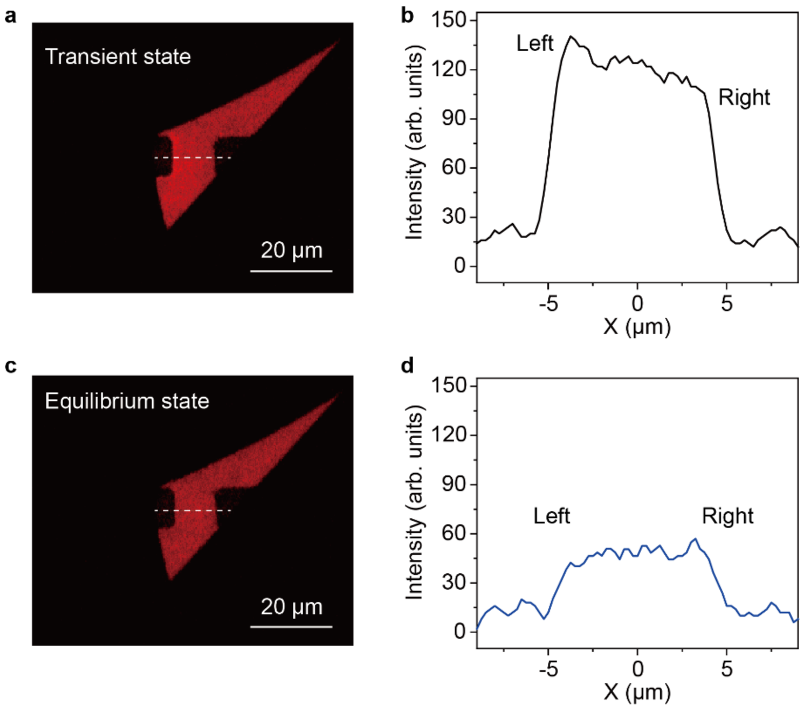

Supplementary Figure 2. (a) Wide-field fluorescence image for a transient state when the bias (+5 V) is just switched on. (b) Intensity profile along the white dashed line in panel a shows decreased intensity from the left to the right. (c) Wide-field fluorescence image at the constant bias window after reaching the equilibrium state (+5 V bias is still applied). (d) Intensity profile along the white dashed line in panel c shows nearly uniform emission intensity from left to the right.

Supplementary Note 3

The reversed exciton flow direction under opposite bias condition has been verified as below. In the monolayer region between the two electrodes, from left to right, we defined 6 positions as P1 to P6 (similar as Fig. 2b in the main text). Under +5 V DC bias, we measured the time trace of excitonic emission (photoluminescence spectra) at

the six positions (Supplementary Figure 3a), which clearly shows that the excitonic modulation (due to hole detrapping) is largest at P1 and decreases from P1 to P6. When we reverse the bias direction, by applying a –5 V DC bias, the opposite happens, with largest modulation switching to P6 (Supplementary Figure 3b).

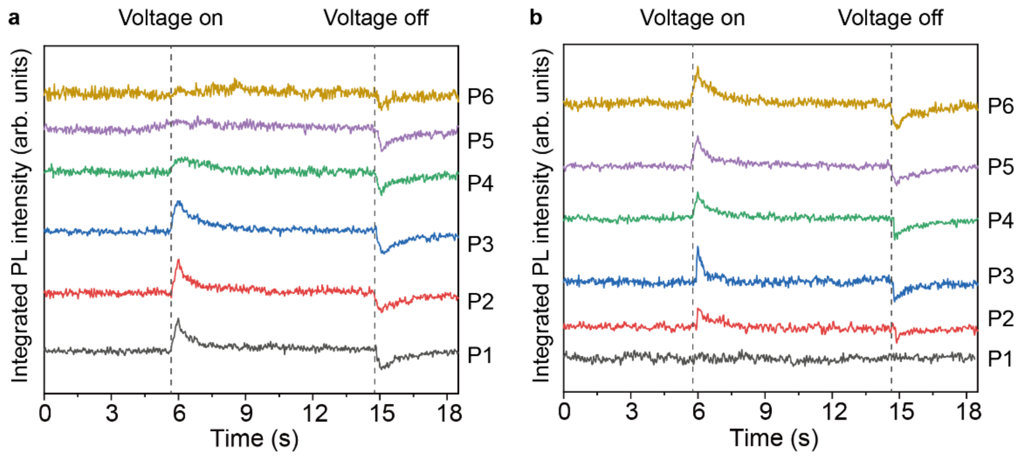

Supplementary Figure 3. Lateral DC bias induced excitonic modulation recorded at different positions (P1 to P6) under excitation of 532 nm laser. (a) Positive bias (+5 V) is applied. (b) Negative bias (–5 V) is applied.

Supplementary Note 4

Supplementary Figure 4 shows the dynamics of excitonic emission measured from different positions (P1 to P6 defined in Fig. 2b) under AC bias with the amplitude of ±5 V and the frequency of 0.1 Hz. The excitonic modulation is sensitive to the distance towards the electrode interface. The modulation depth is larger close to the two Au electrodes (P1 and P6) and smaller near the center (P4). The modulation depth has a

small difference near the two interfaces (e.g., P1 and P6), which may be due to the different contact conditions for the two Au/WS_2 interfaces.

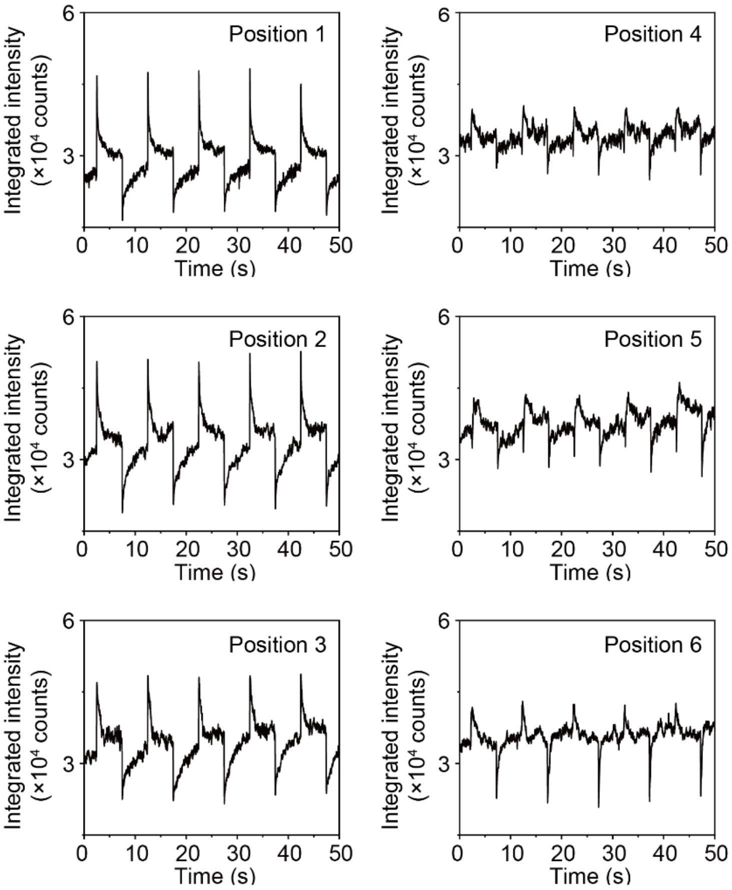

Supplementary Figure 4. Excitonic modulation measured from different positions (P1 to P6 defined in Fig. 2b) under AC bias with the amplitude of ±5 V and the frequency of 0.1 Hz.

Supplementary Note 5

Supplementary Figure 5 shows the correlation between the excitonic intensity modulation and the change in the trion spectral weight at P1 to P6. The black dot represents the excitonic intensity modulation which is calculated based on Fig. 2d by

subtracting the dark state intensity from the bright state intensity at each position. The red line represents the change in trion spectral weight (based on Fig. 2f), which is also calculated as the trion weight difference between the dark and bright states. The good correlation between the two sets of data indicates that the AC bias-induced carrier flow leads to the modulation of excitonic distribution in the monolayer plane.

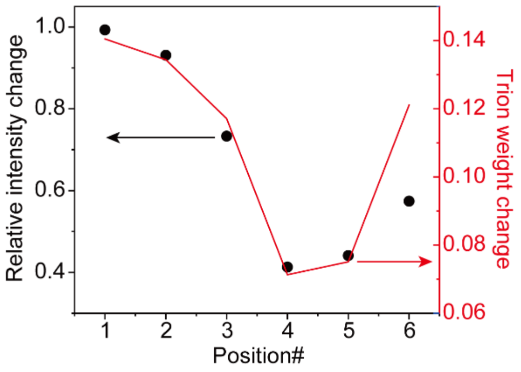

Supplementary Figure 5. Plot of relative intensity change (black dots) and trion weight change (red line) between the bright and dark states at P1 to P6.

Supplementary Note 6

We estimated the local charge carrier density, i.e. the electron density (N_e), of the WS_2 monolayer, based on the following equation:[1,2]

$$\frac{N_e N_X}{N_{X^-}} = \left(\frac{4m_e m_X}{\pi\hbar^2 m_{X^-}}\right) k_B T\, e^{-\frac{E_b}{k_B T}} \quad \text{(Supplementary Eq. 1)}$$

where N_e is the electron density of WS_2, N_X is the concentration of exciton, N_{X^-} is the concentration of trion, m_e is the effective mass of electrons, m_X is the effective mass of excitons, m_{X^-} is the effective mass of trions, $\hbar$ is the reduced Planck constant, k_B is the Boltzmann constant, T is the operation temperature (in our case ~300 K), and E_b is the

trion binding energy (~30 meV). For WS_2, m_e is 0.44 m_0 (and m_h is 0.45 m_0), $m_X = m_e + m_h = 0.89\ m_0$, $m_{X^-} = 2m_e + m_h = 1.33\ m_0$, where m_0 is the mass of a free electron.[2]

The concentrations of excitons and trions are proportional to their PL intensities via Supplementary Equation 2:[2]

$$\frac{I_X}{I_{X^-}} \propto \frac{N_X}{N_{X^-}}\frac{\gamma_X}{\gamma_{X^-}} \qquad \text{(Supplementary Eq. 2)}$$

where I_X is the exciton intensity, I_{X^-} is the trion intensity, γ_X is the radiative decay rate of excitons, γ_{X^-} is the radiative decay rate of trions. Based on Supplementary Equation 1-2, the electron density of WS_2 is written as:

$$N_e = \frac{I_{X^-}}{I_X}\frac{\gamma_X}{\gamma_{X^-}}\left(\frac{4m_e m_X}{\pi\hbar^2 m_{X^-}}\right)k_B T\ e^{-\frac{E_b}{k_B T}} \qquad \text{(Supplementary Eq. 3)}$$

where I_X and I_{X^-} can be obtained from the fitting of the experimental spectra (Figure 2e), and the ratio between γ_X and γ_{X^-} is ~5.08 for WS_2.[2]

With Supplementary Equation 3, we estimated the electron concentration N_e of the bright and dark states for the 6 points (P1 to P6), as shown in Supplementary Figure 6.

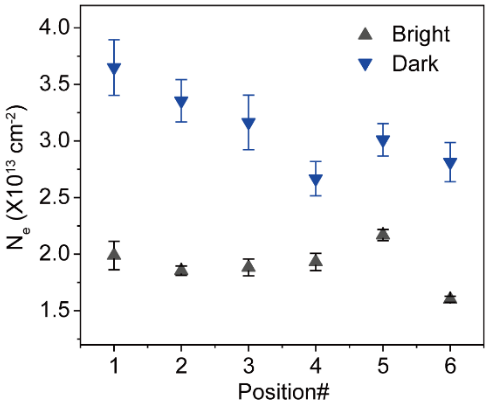

Supplementary Figure 6. Position dependent local electron density N_e for the bright and dark states under lateral AC bias. The error bars represent for the standard deviations from 5 periods under AC bias.

Supplementary Note 7

Below we have analyzed PL spectra at six different positions (P1 to P6) under +5 V DC bias. For each position, we compared PL spectra for the bright state (when bias is switched on) and the dark state (when bias is switched off). We found that similar to the AC bias case, the bright state PL spectrum and the dark state PL spectrum under DC bias also show different intensity ratios of neutral excitons and trions (Supplementary Figure 7a-d). Moreover, when we compare the bright state spectra for the six positions, we observe an increasing trion weight from P1 to P6, corresponding to a flow of trions from left to the right when we switch the bias on. In contrast, when we compare the dark state spectra for the six positions, we visualize a decreasing of trion weight from P1 to P6, corresponding to the flow of trion from right to left when we switch off the bias (Supplementary Figure 7e). The trion weight change, i.e., the difference between bright and dark state, is highest at P1 and lowest at P6 (Supplementary Figure 7f), which corresponds to the largest PL modulation at P1 as shown in Supplementary Figure 3a.

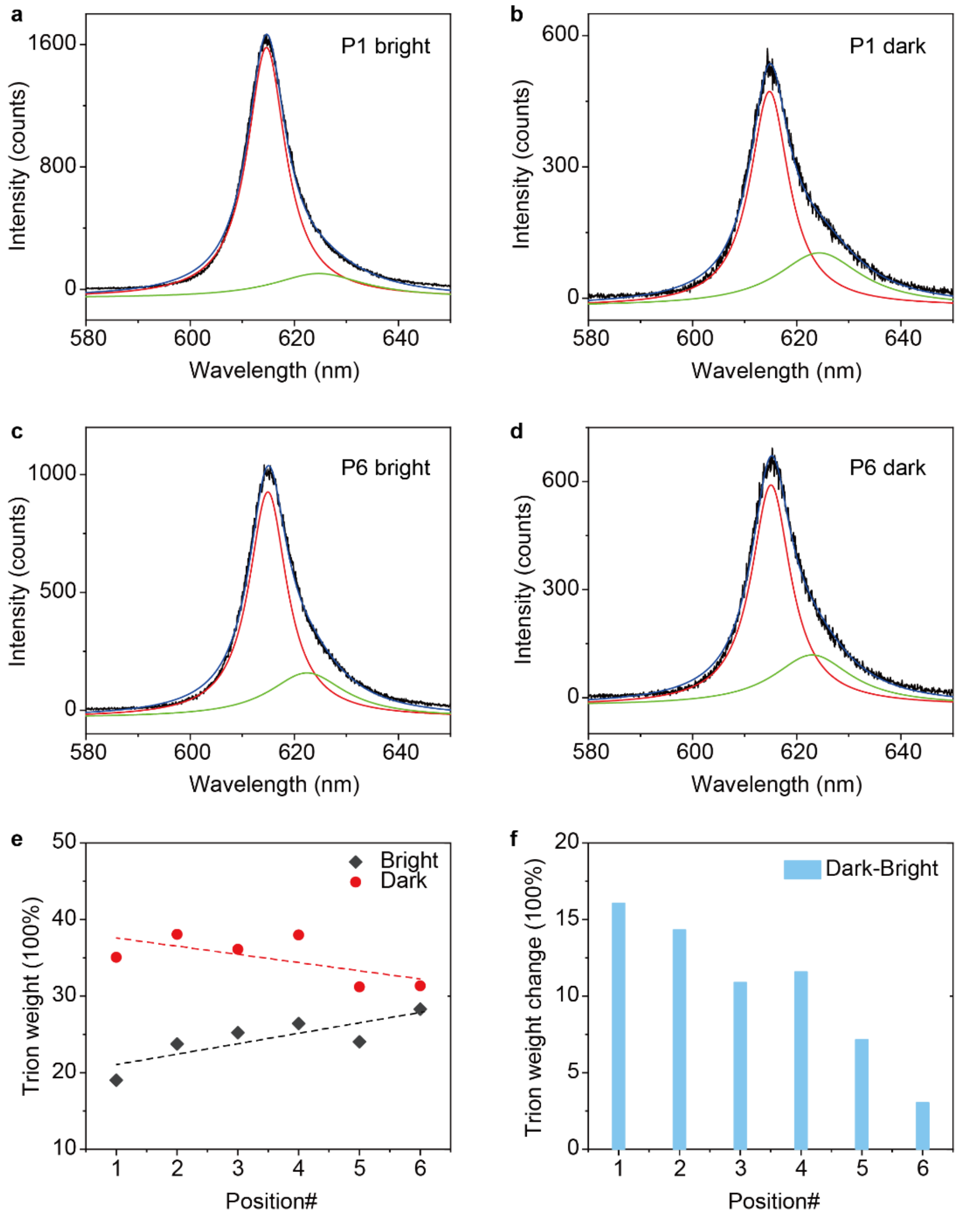

Supplementary Figure 7. PL spectra analysis under lateral DC bias with 532 nm laser excitation. (a,b) Bright state and dark state PL spectra measured at P1 position. (c,d) Bright state and dark state PL spectra measured at P6 position. (e) Position dependent trion spectral weight for the bright and dark states. (f) Plot of trion weight change as a function of measurement position. Positive bias (+5 V) is applied to the left electrode, and the right electrode is grounded.

Supplementary Note 8

Supplementary Figure 8 shows the excitonic modulation (at P1 position) with systematically increasing AC bias amplitude from ± 1 V to ± 10 V with a step of 1 V. The modulation increases quickly at small AC bias amplitude, but tends to saturate at high AC bias amplitude.

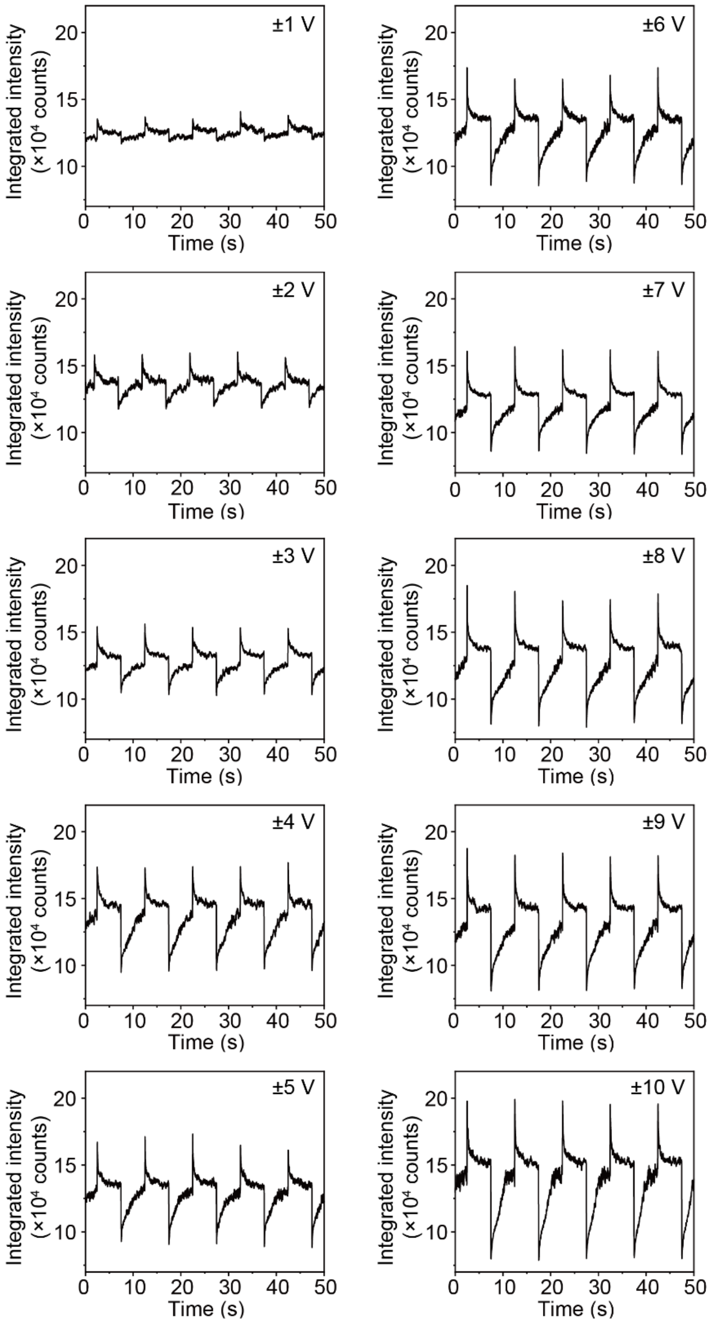

Supplementary Figure 8. Excitonic modulation (at P1) measured under AC bias with the amplitude ranging from ±1 V to ±10 V. The AC frequency is kept as 0.1 Hz.

Supplementary Note 9

To characterize the doping types and carrier concentrations for the different monolayer semiconductors, we have made field-effect transistor (FET) devices using monolayer WS_2, MoS_2 and WSe_2, respectively. Supplementary Figure 9 shows the transfer curves (I_{ds} vs V_{gs}) of the three types of monolayers.

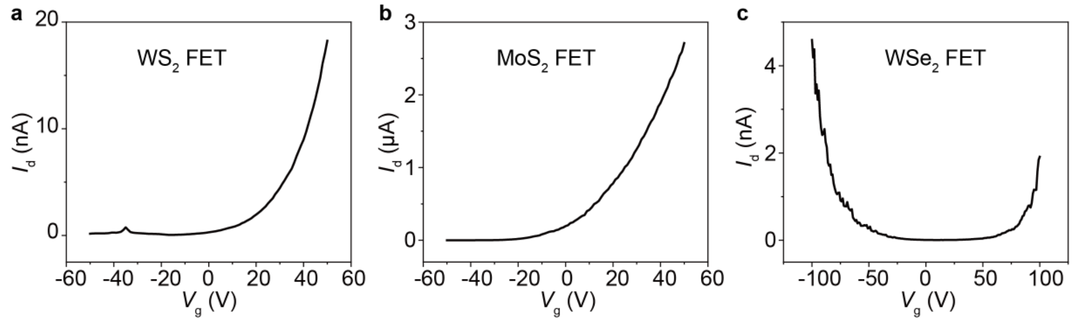

Supplementary Figure 9. Transfer curve of FET devices made from WS_2 monolayer (a), MoS_2 monolayer (b), and WSe_2 monolayer (c).

To calculate the free carrier concentration, we first calculated the mobility of the free carriers with the following equation:[3]

$$I_{ds} = \frac{W}{L}\mu C(V_{gs} - V_{th})V_{ds} \qquad \text{(Supplementary Eq. 4)}$$

where W and L are the width and length of the FET device, μ is the mobility, C is the capacitance per unit area of the FET device, V_{th} is the threshold voltage, V_{ds} is the bias between drain and source. In our case, the ratio W/L is ~50, C is 1.21×10^{-4} F/m^2, V_{ds} is

2 V, and the V_{th} is 34.3 V for the WS_2 case, 17.8 V for the MoS_2 case, and 88.5 V for the WSe_2 case. Further, the free carrier density n can be calculated via Supplementary Equation 5:

$$\sigma = ne\mu \quad \text{(Supplementary Eq. 5)}$$

where σ is the conductance at $V_{gs} = 0$, and e is the unit electron charge. In this way, the derived characteristics of the monolayer FETs is summarized in Supplementary Table 1. Moreover, the calculated electron density based on WS_2 FET devices, is consistent with the estimation based on PL spectra (Supplementary Figure 6). Further, by comparing the static doping of WSe_2 monolayer (7.82×10^{10} cm^{-2}) with the bias induced carrier concentration change (up to 1.5×10^{13} cm^{-2}, as shown in Supplementary Figure 6), the polarity flipping in the WSe_2 monolayer is easy to achieve (Fig. 4b).

Supplementary Table 1

	**WS_2**	**MoS_2**	**WSe_2**
**Doping-type**	n	n	slightly p
**Carrier density *n***	1.56×10^{13} cm^{-2}	8.77×10^{13} cm^{-2}	7.82×10^{10} cm^{-2}

Supplementary Note 10

As shown in Fig. 2c, after the instant excitonic modulation during the bias switching, at the constant bias window, there is a slow relaxation towards equilibrium state. The time scale of the relaxation could be related to the energetics of the interface traps. When the bias is switched on, shallow traps will be released immediately,

generating the largest modulation. However, deep traps are more difficult to be released, which will take longer time. The slowly release of deep traps and the band bending induced carrier drift happens simultaneously, which contribute to the relaxation of the excitonic modulation, until the equilibrium state is achieved. Therefore, the measured relaxation time deviates from sample to sample as it is difficult to control the monolayer/electrode and monolayer/substrate interfaces to be all the same. Based on the data of three different WS_2 junctions fabricated at different times (Supplementary Figure 10), we have fitted the relaxation profiles using a bi-exponential function. The obtained relaxation times range between 0.035 − 0.089 s for t_1 and 0.316 − 0.903 s for t_2.

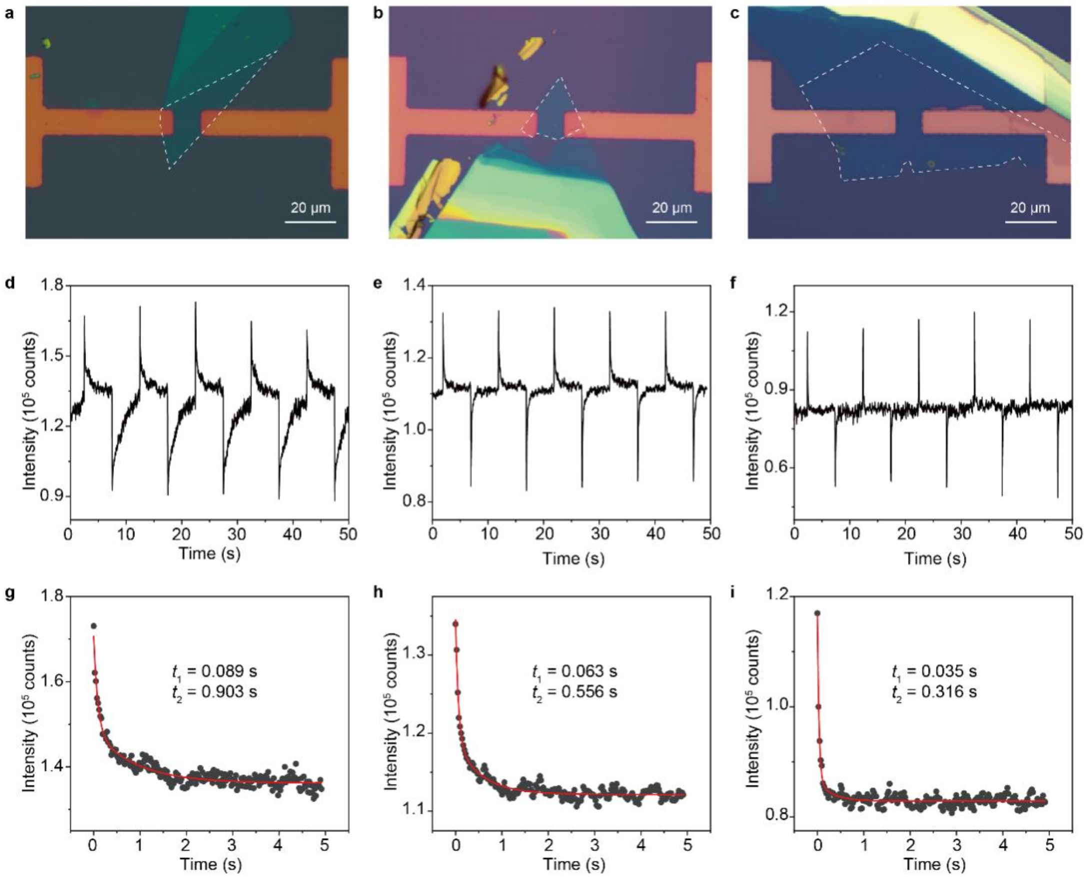

Supplementary Figure 10. (a,b,c) Optical images of three Au-WS_2-Au junctions

fabricated on 285 nm SiO_2/Si substrates. The white dashed lines indicate the boundary of the WS_2 monolayers. (d,e,f) Excitonic modulation in the above three junctions under AC bias of ±5 V and 0.1 Hz. (g,h,i) Fitting of relaxation times for the above three junctions using a bi-exponential decay function.

Supplementary Note 11

The performance of excitonic modulation under high-frequency operation (Fig. 5 in the main text) was investigated using a home-built setup as shown in Supplementary Figure 11. During the measurements, the 532 nm CW laser was used for excitonic excitation, the function generator (FG, Tektronix AFC3011C) was used for high-frequency AC bias or pulse apply, and the single photon avalanche diode (SPAD, MPD PD-100-CTE) was used for excitonic emission detection. The time-resolved excitonic modulation was realized via a time-correlated single-photon counting (TCSPC, Swabian Instruments Time Tagger Ultra) module which synchronized the FG and the SPAD.

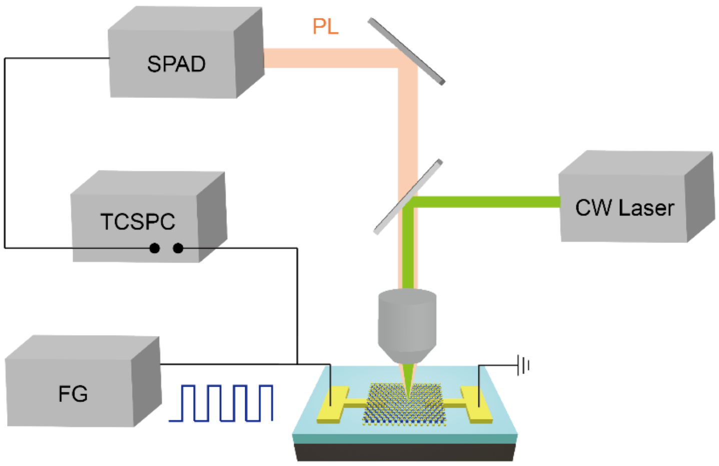

Supplementary Figure 11. Schematic of the setup for high-speed modulation measurements.

Supplementary Note 12

Supplementary Figure 12a shows the optical micrograph of the Au-WS_2-Au junction fabricated on a glass substrate. By applying a lateral AC bias (±8 V, 0.1 Hz) to the Au-WS_2-Au junction on the glass substrate, we also observe bias modulated excitonic emission (Supplementary Figure 12b) with similar dynamics as in Fig. 2c. Supplementary Figure 12c,d show the photoluminescence spectra for the bright and dark states recorded during the AC bias applying. Based on the peak fittings, the trion spectral weight increases from 53.0% for the bright state to 60.3% for the dark state, similar to the case with SiO_2/Si substrates. The bias induced modulation on the glass substrate further supports our trap state model, while the possible floating gate effect which might exist with the 285 nm SiO_2/Si substrate can be completely excluded when using the glass substrate.

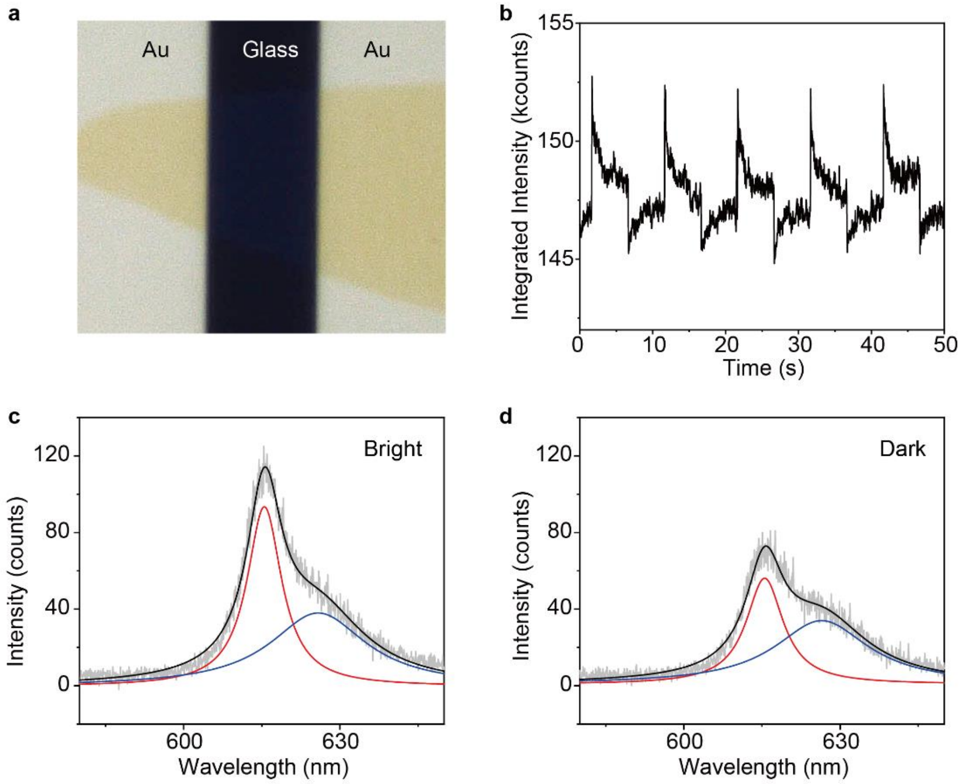

Supplementary Figure 12. Lateral bias induced excitonic modulation with Au-WS_2-Au junction on a glass substrate (with thickness of 0.4 mm). (a) The optical micrograph of the sample. (b) Dynamics of excitonic emission under AC bias with the amplitude of ±8 V and the frequency of 0.1 Hz. (c,d) Emission spectra measured for the bright (c) and dark (d) states.

Supplementary Figure 13 further shows the high-speed modulation measurement on the glass substrate. By excluding the floating gate effect and extra capacitance from the back Si, the modulation frequency of the device is further optimized to 10 MHz with switching time down to 5 ns, which reaches the instrument response limit of our function generator.

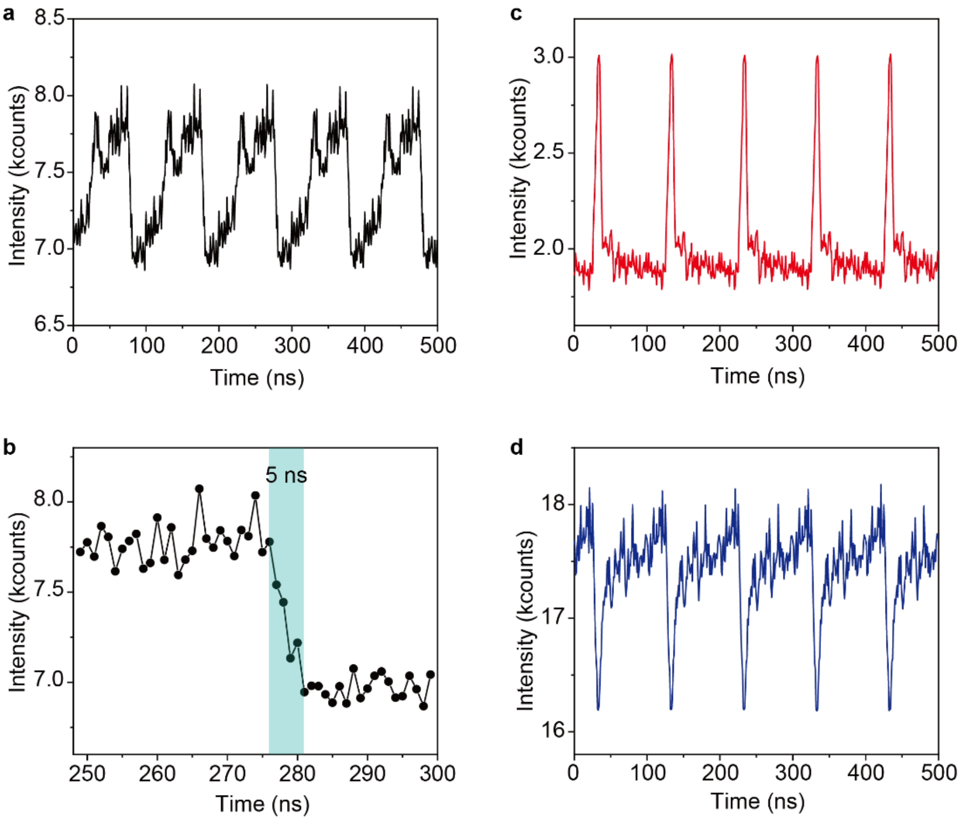

Supplementary Figure 13. High-speed operation with Au-WS_2-Au junction on the glass substrate. (a) Time-resolved excitonic emission of the WS_2 monolayer under AC bias of ±4 V and frequency of 10 MHz. (b) Zoom-in time window from panel a, showing the switching time of 5 ns. (c,d) Time-resolved excitonic modulation induced by the applied electrical pulses measured close to the left (c) and right (d) interfaces. The same electrical pulse (0 to 8 V) was applied with the pulse width of 10 ns and the repetition frequency of 10 MHz.

The ultimate excitonic modulation speed should be limited by the RC delay of the device. By assuming the main capacitance originates from the two Au/WS_2 interfaces, and considering a total Au/WS_2 contact area of 100 μm^2, an average thickness of the van der Waals gap of 1 nm, and an average relative permittivity of $\varepsilon_r = 5$ (materials in

the van der Waals gap could be organic residues, water, and air etc.), the capacitance C of the device will be 4.4×10^{-12} F based on the parallel plate capacitor equation. This corresponds to a cut off frequency $f = 1/2\pi RC \approx 0.7$ GHz and a switching time of 1.4 ns for a device with a resistance R = 50 **Ω**. Based on the above calculation, the experimentally measured switching time of 5 ns hasn't reached the theoretical limitation of our device and is currently limited by our measurement instrument. Using high-resolution lithography technique, it should be possible to further increase the modulation speed by shrinking the Au/WS_2 contact area to the nano-meter scale.

Towards operation at further elevated frequency, we should also consider the stray capacitances from the leads and the impedance matching of all components. Meanwhile, as the modulation of excitonic distributions requires carrier drift in the monolayer plane, the carrier travel time across the monolayer is also important for the high-frequency performance.

Supplementary References

1. J. S. Ross, S. Wu, H. Yu, N. J. Ghimire, A. M. Jones, G. Aivazian, J. Yan, D. G. Mandrus, D. Xiao, W. Yao, X. Xu. Electrical control of neutral and charged excitons in a monolayer semiconductor. *Nat. Commun.* **4**, 1474 (2013).
2. N. Peimyoo, W. Yang, J. Shang, X. Shen, Y. Wang, T. Yu. Chemically driven tunable light emission of charged and neutral excitons in monolayer WS_2. *ACS Nano* **8**, 11320–11329 (2014).

3. J. Wang, Q. Yao, C.-W. Huang, X. Zou, L. Liao, S. Chen, Z. Fan, K. Zhang, W. Wu, X. Xiao, C. Jiang, W.-W. Wu. High mobility MoS_2 transistor with low Schottky barrier contact by using atomic thick h-BN as a tunneling layer. *Adv. Mater*. **28**, 8302–8308 (2016).
