# Supplementary material for: Room-temperature high-speed electrical modulation of excitonic distribution in a monolayer semiconductor
Source: Nat Commun. 2023 Oct 23;14:6701. doi: 10.1038/s41467-023-42568-w (PMC10593816; doi:10.1038/s41467-023-42568-w)
Supplement: Supplementary file 3 — Description of Additional Supplementary Files [file 41467_2023_42568_MOESM3_ESM.docx]

**Description of Additional Supplementary Files**

**Supplementary Movie 1:** the excitonic emission movie with +5V DC bias at ambient condition.

**Supplementary Movie 2:** the excitonic emission movie with -5V DC bias at ambient condition.

**Supplementary Movie 3:** the excitonic emission movie with ±5V AC bias at ambient condition.

**Supplementary Movie 4:** the excitonic emission movie with ±5V AC bias at vacuum condition.
